# Photovoltaic-driven microbial protein production can use land and sunlight more efficiently than conventional crops

**DOI:** 10.1073/pnas.2015025118

**Published:** 2021-06-21

**Authors:** Dorian Leger, Silvio Matassa, Elad Noor, Alon Shepon, Ron Milo, Arren Bar-Even

**Affiliations:** ^a^Systems and Synthetic Metabolism, Max Planck Institute of Molecular Plant Physiology, 14476 Potsdam, Germany;; ^b^Department of Civil, Architectural and Environmental Engineering, University of Naples Federico II, 80125 Naples, Italy;; ^c^Department of Plant and Environmental Sciences, Weizmann Institute of Science, 7610001 Rehovot, Israel;; ^d^Department of Environmental Studies, The Porter School of the Environment and Earth Sciences, Tel Aviv University, Tel Aviv 6997801, Israel;; ^e^The Steinhardt Museum of Natural History, Israel National Center for Biodiversity Studies, Tel Aviv University, Tel Aviv 6997801, Israel

**Keywords:** food security, microbial protein, single-cell protein, electrochemistry, photovoltaics

## Abstract

Population growth and changes in dietary patterns place an ever-growing pressure on the environment. Feeding the world within sustainable boundaries therefore requires revolutionizing the way we harness natural resources. Microbial biomass can be cultivated to yield protein-rich feed and food supplements, collectively termed single-cell protein (SCP). Yet, we still lack a quantitative comparison between traditional agriculture and photovoltaic-driven SCP systems in terms of land use and energetic efficiency. Here, we analyze the energetic efficiency of harnessing solar energy to produce SCP from air and water. Our model includes photovoltaic electricity generation, direct air capture of carbon dioxide, electrosynthesis of an electron donor and/or carbon source for microbial growth (hydrogen, formate, or methanol), microbial cultivation, and the processing of biomass and proteins. We show that, per unit of land, SCP production can reach an over 10-fold higher protein yield and at least twice the caloric yield compared with any staple crop. Altogether, this quantitative analysis offers an assessment of the future potential of photovoltaic-driven microbial foods to supplement conventional agricultural production and support resource-efficient protein supply on a global scale.

## Significance

The cultivation of microbial biomass, which is rich in proteins as well as other nutrients, can play a vital role in achieving food security while mitigating the negative environmental footprint of agriculture. Here, we analyze the efficiency associated with using solar energy for converting atmospheric CO_2_ derived from direct air capture into microbial biomass that can feed humans and animals. We show that the production of microbial foods outperforms agricultural cultivation of staple crops in terms of caloric and protein yields per land area at all relevant solar irradiance levels. These results suggest that microbial foods could substantially contribute to feeding a growing population and can assist in allocating future limited land resources.

Food security is a critical issue that humanity faces in this century. The combined effect of population growth and increasing consumption of animal-based products are projected to cause a surge in demand for food which could severely challenge global production by 2050 (1, 2). Moreover, the regional impacts of climate change pose a threat to future food security in many countries (3). Although, historically, the food supply has expanded alongside increasing demand, major improvements to crops are now slowing as they approach biological constraints (4, 5). At the same time, agricultural land expansion has limited potential to increase supply, since food production currently occupies more than a third of the Earth’s terrestrial surface (6) and already exerts large environmental burdens (7–10). Therefore, addressing food security requires societal changes as well as innovations in the global food system that go beyond conventional agriculture. In the current study, we explore the potential for the cultivation of microbes to help address this global challenge.

Production of nutrient-rich foods derived from microbial biomass, better known as microbial protein or single-cell protein (SCP), offers a promising means to address food security without exacerbating pressure on the environment, as it utilizes water and nitrogen more efficiently than plants (11–14). Several companies are already producing SCP derived from algae, fungi, or bacteria at commercial scale destined for animal or human consumption (15). The feedstock used to cultivate these microbes is typically either agriculturally derived glucose or fossil-derived methane and methanol (11). Yet, a more sustainable alternative, which minimizes reliance on fossil carbons and agricultural land, is to use renewable energy (here, photovoltaics) to convert atmospheric carbon dioxide and water into molecules that can serve as electron donors for microbes (16, 17). Previous studies have considered the land requirements for SCP production using feedstocks derived from agriculture, fossil fuels (12), and, more recently, also renewable energy (18). Nevertheless, a comprehensive assessment of land and energy efficiency of fully photovoltaic-driven microbial food production is still lacking. Focusing on solar energy allows us to compare the potential of food production using microbes against contemporary agriculture on an even playing field, since both technologies rely on the same primary resources (i.e., land, sunlight, water, and fertilizers). More specifically, this study sought to answer how productive photovoltaic-driven SCP (PV-SCP) systems can be in terms of calorie and protein production per unit time and land area in comparison to other SCP systems and to conventional crops, focusing on the effect that solar irradiance has on PV-SCP yields. This quantitative comparison can assist in planning the future allocation of limited land resources toward feed and food production.

We used literature data to calculate the overall efficiency by which solar energy can be harnessed to generate SCP, considering different electron donors and metabolic pathways. We decided to focus on bacteria since they are flexible in their use of feedstock and reach higher protein content than other microorganisms (11). We assumed that all carbon requirements are met by direct air capture of carbon dioxide from the atmosphere (DAC) (19) in order to minimize the reliance on fossil fuels as well as to support a fair comparison with plants. We further took into account other energetic expenditures, such as production of macronutrients for microbial cultivation, bioreactor stirring and cooling, and downstream processing of biomass and proteins. We show that PV-SCP technologies can substantially outperform conventional staple crops in terms of both calorie and protein yield.

## Results

### Energetic Efficiency of SCP Production.

**Fig. 1.**
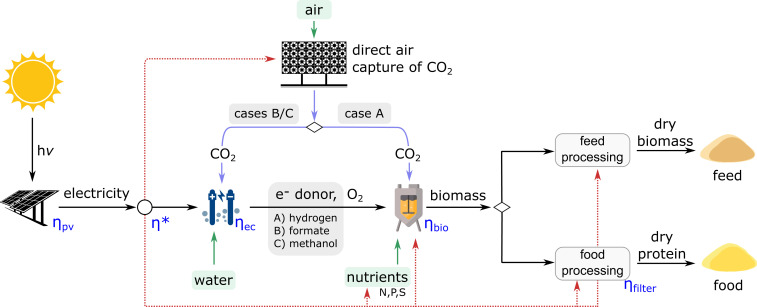
Schematic representation of energy transfer during production of single-cell proteins from solar energy. Each conversion step is associated with an energetic efficiency, η. The effective electricity use efficiency, η*, corresponds to the fraction of electricity used for electrosynthesis of the electron donor. The rest of the electricity (dashed red arrows) is distributed among supporting processes, including DAC of CO_2_, provision of macronutrients, bioreactor operation, and biomass downstream processing. The entry point of CO_2_ in the production chain, depicted by an “exclusive or” rhombus, depends on the choice of the electron donor. When hydrogen serves as the electron donor (case A), concentrated CO_2_ is supplied to the bioreactor along with the H_2_ and O_2_ produced in the electrochemical cell. For the production of formate (case B) and methanol (case C) as electron donors, CO_2_ is supplied to the electrochemical unit, while the only input gas supplied to the bioreactor is oxygen. In each case, we assumed that the oxygen fed into the bioreactor is derived from water splitting (in the electrochemical unit) and that CO_2_ from the bioreactor off-gas is directly recycled with negligible energy cost. Following growth in the bioreactor, the harvested biomass enters downstream processing. Two production scenarios are depicted depending on the desired final product. For the production of animal feed, the feed downstream processing includes only the removal of water, by centrifugation and spray drying, such that all cellular components are retained in the final product. For the production of human food, the food downstream processing includes two additional steps to reject nucleic acids, bead-milling and microfiltration, which discard the nonproteinaceous components from the final product. Hence, the food downstream processing requires additional supporting energy and includes an energy loss step (in the form of discarded biomass), denoted by η_filter_.

We considered a PV-SCP system that converts solar energy into energy stored in food by the following four generalized steps (Fig. 1):

$$\begin{array}{l} Solarenergy\overset{\left(1\right)}{\to }Electricity\overset{\left(2\right)}{\to }ElectronDonor\overset{\left(3\right)}{\to }\\ Biomass\overset{\left(4\right)}{\to }Feed/Food. \end{array}$$

Process (1) corresponds to PV solar farms capturing solar energy and converting it to electricity. Process (2) represents the electrochemical conversion of electrical energy into chemical energy stored in an electron donor and/or carbon source. Process (3) refers to microbial growth which converts the chemical energy from the previous step into chemical energy stored in biomass. Process (4) describes a filtration step whereby nucleotides, fatty acids, and carbohydrates are discarded while only the protein is retained. The removal of nucleic acids is crucial when SCP serves as a human food since in too high of concentrations, their catabolism leads to an accumulation of uric acid, which cannot be easily degraded and can form gout (20). Unlike humans, all farm animals possess the enzyme uricase, which precludes this effect, therefore making nucleic acid removal unnecessary for feed production. Each of these processes is associated with different energetic efficiencies—η_pv_, η_ec_, η_bio_, and η_filter_ (Fig. 1)—which we calculate according to available measurements, as explained in *Methods*. These four steps describe the direct transfer of energy from solar to biochemical storage in food. However, operating the SCP system also requires several electricity inputs not depicted in this linear chain, and we account for all of them by introducing another efficiency term η*, which is described below. For example, η* accounts for the energetic cost of operating DAC which supplies the CO_2_ required at steps (2) or (3).

With respect to the first step, solar energy capture, it is common to report the energetic efficiency of PV solar energy conversion to electricity (η_pv_) as approaching 20%, which represents the solar cell efficiency under standard test conditions (21). However, this neglects numerous practical factors, most importantly PV ground coverage ratio, and losses due to power electronics, solar tracking, inverter, and temperature as well as surface soiling from dust, snow, and other debris (22). To obtain a more realistic view of solar farm efficiency, we used available data from >600 utility-scale sites (Dataset S1A). As explained in *Methods*, we found that η_pv_ ranges between 4.1% and 5.6% (30th to 70th percentiles), considerably lower than the solar cell efficiency. If PV technology is replaced by concentrated solar power (e.g., parabolic trough, power tower, or linear Fresnel reflector), the energetic efficiency is even lower (Dataset S1B); hence, we did not consider this latter technology further.

Following the conversion of solar energy to electricity, electrical energy is converted into chemical energy by producing simple molecules (electron donors), which support microbial growth. To provide a broad perspective on the properties of different electrochemical and biological processes, we considered three electron donors (hydrogen, methanol, and formate) and several microbial assimilation pathways. For the production of all of the three electron donors considered, water is first split and oxidized at an anode to provide electrons and oxygen (O_2_) to the processes. Carbon dioxide (CO_2_), which is the only primary source of carbon in the production process, is obtained via DAC of CO_2_, and, as shown in Fig. 1, there are two possible entry points for CO_2_ (*SI Appendix*).

The energy efficiency associated with the production of an electron donor (η_ec_) is determined by its combustion energy divided by the invested electrical energy. Hydrogen is the most commonly used electron donor, the production of which is relatively efficient via electrolysis, with an η_ec_ of 70% (±5%) (23). Aside from hydrogen, only formate and carbon monoxide (CO) can be directly produced electrochemically at commercially relevant energetic efficiency, Faraday efficiency, and current density (24). We chose to focus on formate as it is miscible, therefore bypassing mass transfer issues that constrain the bio-consumption of low-solubility gaseous compounds such as CO. Moreover, formate can be assimilated into biomass much more efficiently than CO (25). Electrochemical reduction of CO_2_ to formate is characterized by an η_ec_ of 40% (±10%) (24). Electron donors can also be produced from electricity indirectly, whereby electrolysis-derived hydrogen is reacted with CO_2_ to generate a reduced compound. As an example of this approach, we focused on the production of methanol, another miscible compound, as its two-step production from electricity is relatively efficient, with an η_ec_ of 55% (±5%) (24).

As to the microbial assimilation pathways, we focused on bacteria as they are metabolically flexible in terms of electron donor and/or carbon source utilization and can reach higher protein content than algae and yeast (11). We considered only aerobic growth, since anaerobic growth (e.g., acetogenesis) diverts most of the carbon into nonprotein-excreted compounds (e.g., acetate) resulting in low biomass yield. Aerobic growth on H_2_/CO_2_ is mainly supported by the Calvin cycle. Due to the relatively high ATP cost of this pathway, the energetic efficiency associated with this growth is relatively low, η_bio_ of 32% (±5%) (25) (*Methods*). The Calvin cycle can also support growth on formate or methanol—with η_bio_ of 27% (±6%) and 21% (±2%), respectively (25) (*Methods*)—via complete oxidation of these electron donors to support de novo carbon fixation. Microbial growth on formate and methanol can instead proceed via direct assimilation of these carbon sources into biomass, which generally supports a higher energetic efficiency. The serine cycle assimilates formate with a high η_bio_ of 46% (±7%) and methanol with η_bio_ of 35% (±2%) (25) (*Methods*). The Ribulose Monophosphate (RuMP) cycle also supports a high efficiency of methanol assimilation, with η_bio_ of 45% (±3%) (25) (*Methods*). We note that the above figures are derived from laboratory experiments rather than industrial-scale facilities due to lack of data. However, we do not expect drastic changes to η_bio_ at scale since this factor is mostly determined by the stoichiometry of the metabolic pathways in each organism.

We considered two SCP production scenarios leading to an end-product of either feed or food. To produce feed, the entire microbial biomass is dried (by centrifugation and spray-drying) and directly used as feed for animals. To produce food, after dewatering by centrifugation, the proteins are extracted from biomass and purified to discard nucleic acids (e.g., by bead milling followed by microfiltration) and used as a food supplement. As previously mentioned, it is imperative to discard nucleic acids from SCP food products (11). We assumed that the usable cellular protein content lies between 55 to 75% on a dry mass basis, which is characteristic of microorganisms used for SCP production (11, 26). This translates to an energetic efficiency of converting biomass to protein, η_filter_, of 46% to 63% (*Methods*).

A simplistic expression for the overall energetic efficiency of converting solar energy to a feed or food product, η_scp_, should be given by η_pv_ × η_ec_ × η_bio_ × η_filter_ (in which the last term is not needed in the case of animal feed). However, this neglects the energy required to support process operation, that is, the energy not directly transferred from the solar input into the final product and hence that is not accounted for in the above production chain. For the electrochemical process, the value of η_ec_, taken from the literature, already includes the energy required for process operation (*Methods*). Yet, as shown in Fig. 1, five processes have nonnegligible energy inputs that need to be accounted for: DAC of carbon dioxide (Dataset S1C), supply of macronutrients (Dataset S1D), bioreactor operation (Dataset S1D), and downstream processing of biomass (Dataset S1D) (*Methods*). We assumed that PV-derived electricity provides energy for all these processes (27). Hence, as depicted in Fig. 1, the initial electrical energy produced is split between the main production chain and the supporting processes. The fraction of electrical energy used for electrochemistry over the total electrical energy produced is equivalent to an effective electricity use efficiency, which we denote as η* (*Methods*). η* depends on other efficiencies, η_ec_ and η_bio_, and hence is not an independent factor. Note also that 1- η* is thus the fraction of energy diverted to the supporting processes. As shown in Table 1, η* ranges between 64% and 83% (i.e., between 17% and 36% of the electricity produced is consumed by the supporting processes) (*Methods*).

**Table 1.** Summary of the energetic efficiencies (η) associated with the production of SCP for animal feed and human food using different electron donors and metabolic pathways

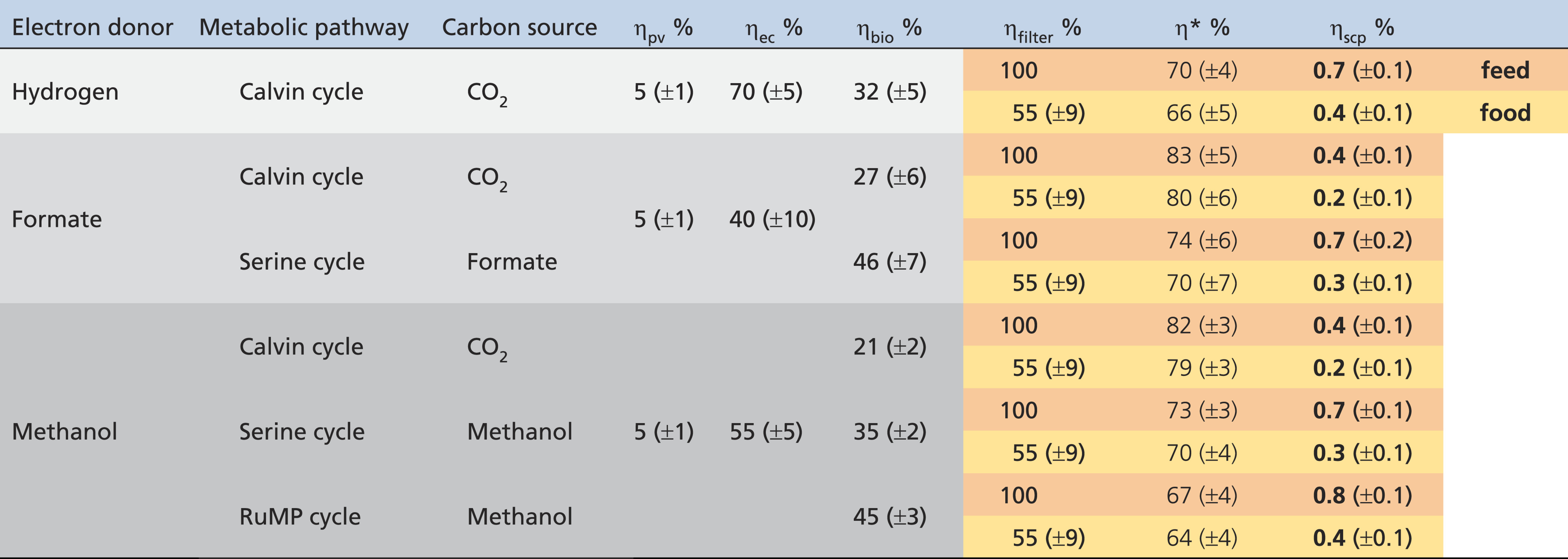

Refer to Fig. 1 and main text for the definitions of the different efficiencies. Each row represents a specific combination of electron donor, metabolic pathway, and type of final product (feed in orange or food in yellow). The overall energy efficiencies, η_scp_, for feed and food production are presented in bold. Processing of biomass to produce feed entails zero loss (η_filter_ = 100%), while food production requires the filtration of all nonprotein mass (therefore, η_filter_ = 55 ± 9%). Values of η_pv_, η_bio_, and η_filter_ are mean estimates (±uncertainty), in which the range represents the 30th and 70th percentiles. The sources for the values of η_ec_ are described in detail in *Methods*. η* and η_scp_ were calculated using Eqs. **5** to **7**, and their uncertainty is derived by error propagation (*Methods*).

The overall energetic efficiency of converting solar energy to the food product, η_scp_, is therefore given by including η* in the previous equation (i.e., η_pv_ × η_ec_ × η_bio_ × η_filter_ × η*). In the case of feed as the final product, η_filter_ is omitted. The energetic efficiency, η_scp_, associated with different SCP production routes is reported in Table 1. Methanol production and assimilation via the RuMP cycle supports the highest η_scp_ (up to 0.8% for feed production and 0.4% for food production) due to the high energetic efficiency of microbial growth on methanol (η_bio_). A similar η_scp_ value can also be achieved by growth on hydrogen, despite its reliance on the relatively inefficient Calvin cycle, due to the high efficiency by which this electron donor is produced electrochemically (η_ec_). Combining the Calvin cycle with other electron donors reduces η_scp_ by half (0.4% for feed and 0.2% for food). Finally, growth via the serine cycle, either on methanol or formate, results in a relatively high η_scp_ (up to 0.7% for feed production and 0.3% for food).

We note that our study included only the operational energy costs of SCP production (i.e., including N, P, and S provision), while energy expenditures for the construction of production facilities were not accounted for. In addition, the DAC process consumes reagents (e.g., sorbents) whose energetic costs of production are difficult to assess due to lack of data (28, 29), and these energy requirements were hence excluded.

### Caloric Yield of Agriculture.

We compared PV-SCP with agriculture in terms of caloric and protein yield per unit land area. We selected the highest protein- and calorie-yielding crops, soybeans and sugar beet respectively, and the seven other staple crops whose total global production by mass is the highest: sugar cane, maize, rice, wheat, potato, cassava, and oil palm. We used a three-year average of the 2017 to 2019 Food and Agriculture Organization Corporate Statistical Database (FAOSTAT) dataset that contains crop mass yields (accounting only for the edible portion of crops) for over 180 nations (Dataset S1E). To obtain a representative value of agricultural productivity that integrates regional differences and avoids outliers, we used production-weighted yields, calculated by averaging each country’s yield weighted by its share of global production. Last, we used the FAO nutritional composition table, which reports the global average protein and kcal content of crops (also only considering the edible portion) to convert crop yields into protein and kcal yields (Dataset S1E). Importantly, food composition tables from various sources (30) suggest that the composition of any particular crop (e.g., due to the use of different cultivars) varies far less compared with regional differences in yield. Therefore, for consistency, we used the single FAO nutritional composition value to convert each crop’s mass yield into protein and caloric yields. We note that each plot of land is assumed to grow one type of crop per year.

Table 2 summarizes the calculated yields of the staple crops. We found soybean to have the highest protein yield, ≈115 g · m^−2^ · y^−1^. The crop with the highest caloric yield is sugar beet, ≈4,520 kcal · m^−2^ · y^−1^, followed by maize and oil palm, ≈2,640 and ≈2,650 kcal · m^−2^ · y^−1^_,_ respectively. All other crops in Table 2 have lower energy and protein yields, and therefore, our conclusions apply to them as well.

**Table 2.** Summary of cultivation yields of the world’s nine most produced staple crops

Crop	Yield (weighted average)		World total	
	Protein [g · m^−2^ · y^−1^]	Energy [kcal · m^−2^ · y^−1^]	Area [Mha]	Fresh weight [Mtons · y^−1^]
Soybeans	115	1,010	123	346
Sugar beet	84	4,520	5	289
Maize	71	2,640	195	1,133
Wheat	51	1,390	216	757
Potatoes	40	1,680	17	368
Rice, paddy	31	1,450	157	713
Sugar cane	15	2,220	26	1,880
Cassava	13	1,560	27	291
Oil palm	5	2,650	28	405

The yield reported corresponds to a weighted average in which each nation’s yield is weighted by its contribution to the global output of the crop (Dataset S1E). Kilocalorie and protein yields are calculated using the FAO nutritional composition table (Dataset S1E).

We emphasize that these crops have varied roles in society, which may make SCP either more or less suitable to replace them. For example, soybean is primarily cultivated for the production of protein-rich animal feed and is well suited for substitution by SCP production (12). As sugar beet is primarily cultivated to produce sucrose and is rarely used as a stand-alone food source, it may be more difficult to substitute with SCP, though it too plays a role in feed production. Sugar beet exhibits relatively high protein yield due to its high biomass yield per unit land, yet its low protein content (≈1.3%) makes it impractical as a source of protein additive for animal feed. Maize is grown for food, feed, and bioenergy production. Oil palm is a major source of fatty acids in the food industry, but since in this analysis we only consider protein for food, it is not a prime candidate for replacement with SCP (31).

### Caloric Yield of PV-SCP Production Can Surpass That of Crops.

To compare microbial foods with agriculture, we calculated the caloric yield Y_cal_ of PV-SCP production in units of kilocalories per square meter per year. Y_cal_ equals the previously calculated η_scp_ multiplied by the relevant irradiance levels (I), for which we assumed a global range of 700 to 2,700 kWh · m^−2^ · y^−1^. However, we identified a statistically significant (*P* value < 0.001) negative correlation between irradiance and the energetic efficiency of utility-scale solar farms (η_pv_), which might be attributed to the known negative correlation between temperature and cell voltage (32). We derived a fitted unitless “correction function,” f_C_ = 1.6 – I/(2,800 kWh · m^−2^ · y^−1^) (*Methods*) to account for the effect of irradiance on solar farm efficiency. Using this correction, the predicted η_pv_ efficiency at 1,000 kWh · m^−2^ · y^−1^ is 6.0%, while at 2,500 kWh · m^−2^ · y^−1^, it is reduced to 3.4%. Altogether, Y_cal_ = I × f_C_ × η_scp_. We note again that in feed production, Y_cal_ refers to the energy stored in all cellular components, including proteins, carbohydrates, and lipids, while for food production, Y_cal_ refers only to the energy stored in the protein fraction of the microbial biomass.

**Fig. 2.**
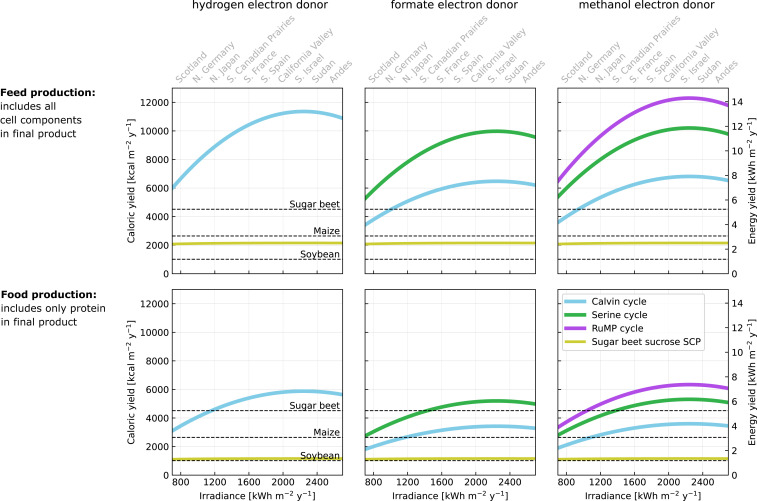
Caloric yield of PV-SCP production for feed (*Top*) or food (*Bottom*) as a function of irradiance. We analyzed different electron donors and assimilation pathways in comparison with three staple crops with the highest caloric or protein yields (Table 2). For feed production, all cellular components are included in the final product, and hence, a higher energy yield is achieved. For food production, only proteins are retained in the final product, leading to a lower energy yield. Furthermore, the latter scenario requires more downstream processing energy. The curves correspond to the mean caloric yields, as explained in *Methods*. The yellow line corresponds to the cultivation of microbes on sucrose extracted from sugar beet. Crop yields correspond to the production-weighted global average yield (Table 2). We note that as crop yields depend on irradiance, the values shown here should be regarded only as a reference to which SCP production is compared. World regions mentioned above the graphs correspond to areas representative of the irradiance levels.

Fig. 2 shows the expected Y_cal_ for SCP production under the two scenarios, feed (above) and food (below), for different combinations of electron donor and assimilation pathways. Our results indicate that PV-SCP production can support higher energy yield than agriculture in both scenarios (i.e., when considering all cellular components or only the protein content). Specifically, at irradiance >1,600 kWh · m^−2^ · y^−1^, the PV-SCP technology can double, triple, or, in some cases, even quadruple agricultural caloric yield for the production of animal feed. It can also support a caloric yield for human food that is more than double that of cereals such as maize, rice, and wheat. A practical alternative SCP production route that bypasses the need for electrochemical reduction or specialized microorganisms (i.e., those that can utilize hydrogen/formate/methanol) is growing heterotrophic microbes on sugars derived from agriculture. For example, Quorn manufactures food products in this way by cultivating the fungus *Fusarium venenatum* on wheat-derived glucose (33). Here, we used sugar beet–fed SCP production as a reference to compare PV-SCP yields against agricultural-based SCP production. As shown in Fig. 2, the energy yield obtained in this case (yellow strip), too, is considerably lower than that of PV-SCP.

### Protein Yield of PV-SCP Production Substantially Surpasses That of Crops.

**Fig. 3.**
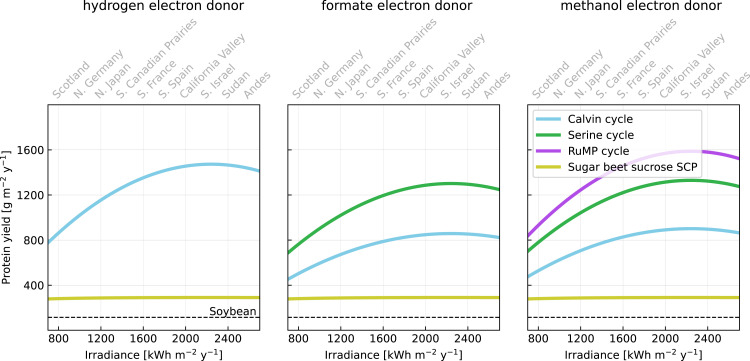
Protein yield of PV-SCP production as a function of irradiance. We analyzed different electron donors and assimilation pathways in comparison with soybean—the staple crop displaying the highest protein yield. The curves correspond to the mean protein yield, as explained in *Methods*. The production-weighted global average protein yield of soybean cultivation is displayed as a horizontal line. We note that as crop yields depend on irradiance, the value shown here should be regarded only as a reference to which SCP production is compared. World regions mentioned above the graphs correspond to areas representative of the irradiance levels.

Next, we focused on the production of proteins and compared the protein yield obtained from agricultural sources (Table 2) with that expected from PV-SCP production. We converted the caloric yield obtained from the previous analysis on food production to units of mass in grams protein (*Methods*). As demonstrated in Fig. 3, the protein yield of PV-SCP is much higher than that of soybean—the crop displaying the highest protein yield (≈115 g · m^−2^ · y^−1^)—regardless of the electron donor and metabolic pathway used. Growth on hydrogen (via the Calvin cycle), as well as growth on methanol or formate via the serine and RuMP cycles, respectively, could support an order of magnitude higher protein yield (>1,200 g · m^−2^ · y^−1^). Although data about the efficiency of converting solar energy into food for crops is not globally available, we calculated this efficiency for maize, sugar beet, and soybean for several countries (Dataset S1F and *SI Appendix*). We observed that the advantage of PV-SCP in terms of efficiency corroborates our findings in terms of caloric and protein yields. Even the cultivation of microbes on sucrose extracted from sugar beet (yellow strip) supports substantially higher protein yield than agriculture, though not as high as PV-SCP.

**Fig. 4.**
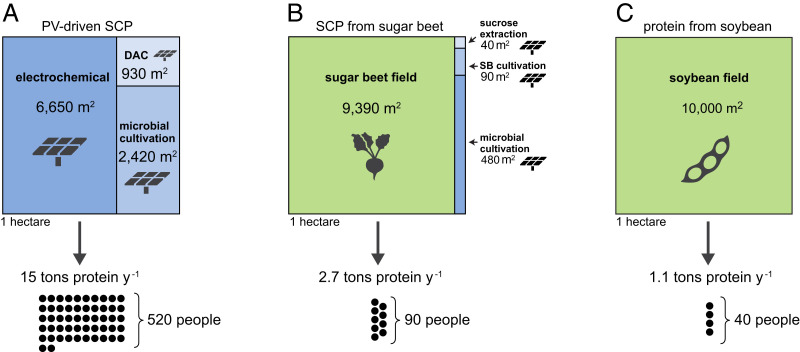
Division of land for the production of nutritional protein, using three different production strategies. The protein yields and amount of people that could be fed from 1 ha are shown at the bottom. (*A*) Photovoltaic-driven production of SCP with hydrogen as the electron donor, assuming an irradiance of 2,000 kWh · m^−2^ · y^−1^. (*B*) Sucrose extracted from sugar beet used to cultivate microbes for the production of SCP, assuming an irradiance of 2,000 kWh · m^−2^ · y^−1^. (*C*) Proteins from the cultivation of soybean, the staple crop with the highest protein yield, assuming a yield of 115 g protein · m^−2^ · y^−1^ (a representative average value based on FAO data). The gray icons correspond to the specific allocation of each plot. DAC corresponds to direct air capture of CO_2_. A daily protein consumption of 80 grams per person is assumed.

To give a more tangible picture of the energy demand for PV-SCP, we analyzed how a land area of one hectare (10,000 m^2^) that receives 2,000 kWh · m^−2^ · y^−1^ of solar energy should be divided so as to support the different components involved in PV-SCP. As shown in Fig. 4*A*, when using hydrogen as an electron donor, about 6,650 m^2^ should be devoted to PV electricity production for water splitting. The energy required for microbial cultivation (which includes the provision of nutrients, bioreactor operation, and biomass downstream processing) and DAC of CO_2_ would require ≈2,400 m^2^ and ≈900 m^2^ of PV arrays, respectively. We note that the land area required for the production facilities (e.g., electrolyzer, bioreactor, and DAC of CO_2_) is negligible compared with the area of the PV arrays needed to energize the processes and hence were not included in the calculation (12, 27). Overall, one hectare devoted to PV-SCP in this design could supply the protein requirement for more than 500 people (15 tons of protein per year; Fig. 4*A*; *Methods*).

To produce SCP from sucrose that is extracted from sugar beet, ≈9,400 m^2^ should be allocated for the cultivation of the sugar beet, while only ≈600 m^2^ would be covered with PV arrays to energize microbial cultivation, sugar beet cultivation, and sucrose extraction (Fig. 4*B*). Such a system is interesting as a simple intermediate solution which uses only mature technologies and does not rely on DAC or electrolysis. Furthermore, it can be viewed as a benchmark for existing microbial food facilities such as Quorn. For simplicity, here we assumed that the varied types of energy used in sugar beet cultivation are equivalent to an electrical energy input that is provided by PV. This production strategy could supply protein for 90 people (2.7 tons of protein per year). Finally, cultivation of soybean would produce only enough protein to feed 40 people per hectare (1.1 tons of protein per year; Fig. 4*C*), which is about a tenth of the people that can potentially be nourished by PV-SCP. The energy inputs into processes supporting soybean cultivation (e.g., for fuel and fertilizers) were not considered here, as they are not met by PV in conventional agriculture. Nonetheless, their inclusion would further lower the yield of soybean cultivation.

## Discussion

We compared PV-SCP production and agriculture according to the expected annual nutritional yield per land area. Our analysis revealed that under the relevant irradiance levels, the total caloric yield obtained from PV-SCP production could be higher than that achieved by the agricultural cultivation of all staple crops. The potential advantage of PV-SCP production becomes even more pronounced when considering its protein yield, which is an order of magnitude higher than that of soybean (by far the highest protein-yielding staple crop; Table 2). We further analyzed an alternative SCP production system which relies on agricultural sugars to feed microbes—rather than electrochemistry, DAC, and specialized metabolic pathways. Although this system yields at least twice the amount of protein compared with soybean, it is still outperformed by PV-SCP by at least fivefold (Fig. 4). These results highlight the potential of next-generation SCP systems as highly land- and energy-efficient alternatives to plant-based protein.

Our study indicates that the choice of electron donor and metabolic pathway can affect the overall yields of PV-SCP production. We find that both hydrogen and methanol (in combination with the Calvin and RuMP cycle, respectively) can achieve the highest energetic efficiencies. On the other hand, hydrogen has very low solubility in water, limiting its transfer to the growing cells and thus constraining cell titer and productivity (25). Furthermore, its dissolution into the growth media requires intense mechanical stirring which generates additional heat and increases bioreactor cooling costs (34). Methanol and formate, which are liquid in atmospheric conditions and completely water miscible, bypass these constraints and can be easily stored and transported—unlike hydrogen and oxygen which require the implementation of expensive safety equipment and procedures. This facilitates the spatial and temporal decoupling of the electrochemical process from microbial cultivation, thus insulating it from the intermittent nature of renewable energy. The downside of using methanol and formate is their tendency to inhibit cell growth at high concentrations, which mandates regulated cultivation regime and could hinder high cell titers and productivities (25).

The possible order-of-magnitude improvement in protein yield stems from at least four facets of the PV-SCP production process: 1) the higher protein content of SCP biomass (>65%) compared with soybean grain (≈38%) (Dataset S1E), 2) greater allocation toward the edible portion of SCP biomass (55 to 75%) (11) compared with soybean (≈34%) (*SI Appendix*), 3) the higher energetic efficiency by which silicon-based PV cells combined with electrochemistry can convert light energy into energy stored in chemical bonds as compared with plant-based photosynthesis (21), and 4) the seasonality of plant growth, such that a large fraction of annual sunlight is not intercepted by crops.

The relevance of our results is strengthened by our use of empirical data to estimate the efficiency of the production steps (i.e., rather than relying on theoretical estimations of process parameters). For example, while it is commonly assumed that the conversion of solar energy to electricity can operate at close to the solar cell efficiency (≈20%), empirical data clearly show that the actual annual efficiencies at utility scale are much lower (≈5%). On the other hand, the nascent power-to-food SCP processes present pragmatic avenues for further efficiency improvements. For example, several emerging and maturing PV technologies (so-called third-generation PV) can surpass the Shockley–Queisser limit of 34% efficiency and have the potential to dramatically increase η_pv_ (35, 36). Multijunction PV has a theoretical upper limit of 68% for nonconcentrated sunlight (36). As there is a strong market incentive to improve PV efficiency outside of food production, such technological gains are expected to develop rapidly in the coming decades. In addition, the emergent “artificial photosynthesis” technologies which directly convert solar energy to chemical energy through photoelectrochemical reactions and particulate photocatalysts may one day improve the efficiency of harnessing solar energy for SCP production by combining η_pv_ and η_ec_ into a single step (37, 38). Regarding η_ec_, we note that while water electrolysis is a relatively mature technology, electrosynthesis of formate and methanol are at an early stage of development. Hence, improvements in these technologies are expected to boost their η_ec_.

Similarly, on the biological side, recent progress in designing and engineering synthetic metabolic pathways for microbial assimilation of formate and methanol with higher energetic efficiency could assist in boosting η_bio_ (39). In addition, focusing on microbes with high protein content or, alternatively, engineering high protein content in industrial model organisms could increase η_filter_. Although crops may also be bred to improve yields, the gains in productivity throughout the Green Revolution were chiefly driven by higher partitioning of biomass to edible grain, and these are now near their upper theoretical limit (4). Hence, the next major improvements to plant yields are likely to be unlocked by profound redesigns of plant biology (e.g., by redesigning the photosynthetic apparatus to capture a larger spectrum of solar energy) or improving carbon fixation (21). Additionally, agriculture may become more sustainable if plants are engineered for more effective nitrogen assimilation, for instance, by enabling symbiosis between nitrogen fixing bacteria and cereal crops or by directly introducing nitrogenase into plants (with these latter two having the added environmental benefits of decreasing the nitrogen pollution and greenhouse gas [GHG] emissions associated with the use and synthesis of N-fertilizers) (4, 40–42). Despite the potential that genetically engineered crops and bacteria offer in terms of efficiency and mitigation of environmental impact, there is growing demand for genetically modified organism (GMO)-free products. Therefore, we focused only on naturally occurring microorganisms to demonstrate the relative benefits of SCP. It is worth noting, however, that many of the crops considered in our comparison are in fact typically cultivated as GMOs, especially soybean and US maize (43, 44).

Ultimately, wide-scale adoption of SCP technologies will be mainly contingent upon their economic competitiveness in the food and feed markets. Recent studies have shown that various SCP production pathways, including hydrogen- and sugar-based systems, could achieve costs that are in line with high-quality feed additives of animal origin such as fishmeal (45). We created preliminary cost estimates for the PV-SCP pathways envisioned in the present study, involving PV electricity, DAC of CO_2_, and green ammonia synthesis. Although our estimates do not represent a comprehensive techno-economic analysis, they enabled us to qualitatively compare the various PV-SCP pathways and draw general conclusions about the relative contribution of the main cost components. For instance, we find that the majority of costs are associated with the production of the electron donors. Our estimates for hydrogen-based SCP feed (≈$2.6 per kg-dw-biomass) are in agreement with findings from the above-mentioned studies (45) and show a similar range to that of methanol-based SCP feed produced through the RuMP metabolic pathway (≈$2.8 per kg-dw-biomass), whereas formate-based production is considerably more expensive (*SI Appendix*, Table S1 and Dataset S1G). When expressed on a per unit protein basis, these production costs are about $4 to $5 per kg-protein, which reveals costs higher than the market price of fishmeal (≈$2.5 per kg-protein) and soybean meal (≈$1 per kg-protein) (*SI Appendix*, Table S1). The food market, on the other hand, is characterized by higher market prices for protein, which could make SCP’s production costs viable (46) (*SI Appendix*, Table S1). For instance, the prices of commonly used whey protein ($7 per kg-protein) and vegetable proteins such as pea ($5 per kg-protein) are on par with our projected PV-SCP production costs. When compared with the prices of mycoprotein ($13 per kg-protein), the main building block of Quorn products, or with emerging alternatives such as insects ($40 per kg-protein), PV-SCP could more easily become price competitive.

We note that the range of energy efficiencies in PV-SCP discussed above is in part a function of technology choices and capital expenses, with larger investments unlocking higher efficiencies. Future trends in the availability of arable land may have a substantial effect on the economics of efficient but expensive solar harnessing technologies by making land-inefficient technology choices increasingly unprofitable.

Clearly, there are other challenges for marketing SCP as human food beyond just its price, such as meeting safety standards and demonstrating health benefits, overcoming regulatory hurdles, promoting consumer acceptance, and improving palatability. Although these market trends are difficult to predict, consumer acceptance does seem achievable since fermentation by bacteria and fungi has traditionally been used for processing many types of food (e.g., *Saccharomyces cerevisiae* for bread, *Lactococcus* for dairy products, and *Aspergillus oryzae* for soy sauce). Furthermore, contemporary companies such as Quorn have demonstrated successful marketing campaigns for fungi-based products (11). From a nutritional perspective, bacterial SCP has a high-quality amino acid profile, which is richer in essential amino acids than soymeal and close to the quality of fishmeal (47). Bacterial SCP is also rich in B vitamins (B1, B2, B3, and B8) (47) and can serve as a valuable complement for plant-based diets which tend to lack vitamin B3 (48). Furthermore, bacterial SCP provides an array of key minerals, including iron, zinc, calcium, phosphorus, potassium, sodium, magnesium, copper, and manganese (47), which encompass multiple micronutrients known to be deficient in the diets of several large populations worldwide (49). Follow-up studies should look further into which commodities are most readily substituted by SCP, incorporating a quantitative analysis of the micronutrients, as well as economic and societal considerations (e.g., cultural and taste preferences). Finally, microbial cultivation can be used for the production of various other commodities, beyond protein. For instance, some natural bacteria produce large amounts of palmitic and oleic acids (50), which are the primary components of palm oil. Hence, PV-driven microbial biomass could potentially also alleviate the environmental pressure of oil crops (51).

In all aspects except price, it seems that SCP is better suited to play a major role in substituting protein-rich animal feeds such as soymeal and fishmeal (12) rather than substituting human foods. Market penetration of SCP for feed is already validated by the success of SCP feed manufacturers such as Unibio and Calysta (11). Notably, several studies have shown that SCP provides health benefits to animals when included at up to ≈50% of their diet, particularly in the case of aquaculture (15, 52, 53). After extensive trials, the European Union approved bacterial SCP feeds (grown on natural gas) in 1995 as nutritional support for the diets of pigs, veal calves, and salmon (at levels of up to 8%, 8%, and 33% of their total feed, respectively) (47). Moreover, due to the rapid growth of the aquaculture sector and concomitant concerns of overfishing for fishmeal, there is an increasing commercial interest in microbial proteins destined for aquaculture (15, 54). Although the projected PV-SCP production prices are currently higher than soymeal or fishmeal, SCP could become more economically viable if the externalized costs linked to pollution, GHG, and ecosystem disruptions caused by extensive soy cultivation and fishing were reflected in prices by policy incentives (45). Furthermore, GMO-based technologies could open new markets by lowering the production costs and providing customized feed-additives (55), yet this may in turn prevent the sale of SCP in countries that heavily regulate GMOs.

Further research is needed to broaden our understanding of the overall impacts of substituting agricultural crops with PV-SCP. To our knowledge, no SCP studies have yet considered the life cycle of PV arrays within their analysis (56). Clearly, sustainable production and recycling of PV arrays are vital for PV-SCP to be environmentally friendly (57).

Yet the technology shows promise, as previous reports demonstrate that SCP uses water and nitrogen resources far more efficiently than crops (54, 58). Modern agriculture relies on large inputs of chemical nitrogen fertilizers to support crop productivity due to low N uptake by plants (≈50%) (59). In contrast, in-reactor SCP production can be tuned to use all the supplied reactive N, thereby performing conversion of inorganic N to edible protein without losses (59). Furthermore, plants and animals use ≈100 and ≈10,000 times more water than SCP (54, 60). This makes SCP an attractive option for regions facing both water and food security risks.

Taken together, production of SCP provides a compelling alternative for the sustainable supply of nutrients, which can rival and outperform contemporary agriculture in many aspects. Our analysis indicates that diverting land resources toward SCP production can help close the approaching “protein gap” (1) while curtailing further agricultural land expansion, thus safeguarding biodiversity and the carbon sink potential of forests and grasslands. Importantly, instead of directly competing for land resources, PV-SCP production can rely on land unsuitable for agricultural use (including urban areas and deserts), rendering a flexible and logistically efficient production process (58). In addition, PV-SCP production is considered to be climate independent and hence can mitigate food supply risks posed by climate change (56). Although SCP currently faces some challenges such as consumer acceptance in the food market or the competitive pricing of the feed market, its commercial viability is likely to improve as land resources become scarce and conventional food sources become increasingly expensive and unsustainable. Furthermore, the high resource efficiency that characterizes SCP production in terms of energy, land, water, and nutrient use also make it a prime candidate to support food production in future long-term missions in space and permanent settlements on extraterrestrial bodies (61, 62).

## Methods

### Solar-to-Electricity Energy Efficiency.

The first step of the solar-to-feed/food process is the conversion of solar energy to electricity. We calculated the energy efficiency of this process, η_pv_, using available information on 628 utility-scale (>1 ha) PV solar farms (including 347 from the United States, 73 from Japan, 35 from France, and 28 from China; Dataset S1A):

$$\eta_{\text{pv}}=\frac{{\text{E}}_{\text{out}}}{{\text{I}}_{\text{in}}\times \text{A}},$$ [1]

where E_*out*_ is the annual electrical energy output of a solar farm, A is the total area of the solar farm, and I_*in*_ is the local annual irradiance energy incident per unit area. Electrical output and solar farm size were provided by Wiki-Solar as annual design output and, when available, by the US Energy Information Administration as average annual electrical output (Dataset S1A). Annual irradiance, for which we used Global Horizontal Irradiance, was determined using Solargis Prospect tool (63). The median η_pv_ was 4.9%, which implicitly incorporates several factors. We defined the lower and upper bounds of η_pv_ as the 30th and 70th percentile: 4.1% and 5.6%. We note that relatively low η_pv_ values, compared with ≈20% solar cell efficiency, are mainly attributed to ≈50% solar panel ground coverage ratio (to prevent interrow shading) (64, 65).

The median energetic efficiency associated with concentrated solar power is 4.1% (Dataset S1B), that is, lower than that of photovoltaic farms, and hence was not further considered.

### Electricity-to-Electron Donor Energy Efficiency.

The energetic efficiency of converting electrical energy to chemical energy stored in an electron donor molecule (i.e., microbial feedstock) is described by η_ec_:

$$\eta_{\text{ec}}=Y_{\text{ED}}\times \mathrm{\Delta H}{{}^{\circ}}_{\text{ED}},$$ [2]

where Y_ED_ is the electron donor yield per unit of input electrical energy and ΔH°_ED_ is the electron donor heat of combustion on a lower heating value basis (reflecting stored energy). $\eta_{\text{ec}}^{H}$, $\eta_{\text{ec}}^{F}$, and $\eta_{\text{ec}}^{M}$ correspond, respectively, to the energy efficiency of producing hydrogen, formate, and methanol. Based on a literature survey, $65\%<\eta_{\text{ec}}^{H}<75\%$ (66), $30\%<\eta_{\text{ec}}^{F}<50\%$ (16, 67–69), and $50\%<\eta_{\text{ec}}^{M}<60\%\;$ (24, 70). We note that $\eta_{\text{ec}}^{H}$ and $\eta_{\text{ec}}^{M}$ include also peripheral energy requirements, such as heating, cooling, pumping, compression, airflow, etc. For formate electrosynthesis $\left(\eta_{\text{ec}}^{F}\right)$, no such data on peripheral energy requirements is available; hence, we used the relatively wide range given above for its energetic efficiency.

In each case, water supplies electrons to produce the electron donors.

$$\begin{array}{l} 2H_{2}\text{O}\to 2{\text{H}}_{2}+{\text{O}}_{2\;}\left(\text{hydrogen}\right)\\ \;2{\text{H}}_{2}\text{O}+2{\text{CO}}_{2}\to 2\text{HCOOH}+{\text{O}}_{2}\left(\text{formic}\;\text{acid}\right)\\ 2{\text{H}}_{2}\text{O}+{\text{CO}}_{2}\to {\text{H}}_{3}\text{COH}+1.5{\text{O}}_{2}\left(\text{methanol}\right). \end{array}$$

For hydrogen and formate synthesis, 1 mol of water is required per mol of electron donor produced. Methanol synthesis requires two steps. In the first, 3 mols of H_2_O are electrolyzed to produce 3 mols of H_2_ and 1.5 mols of O_2_. Then, the H_2_ stream is channeled to a separate catalytic reactor and reacts with CO_2_ to generate 1 mol of methanol while also regenerating 1 mol of H_2_O. In the overall reaction, 2 mols of water are required to produce 1 mol of methanol.

### Electron Donor-to-Biomass Energy Efficiency.

The efficiency with which microorganisms convert the chemical energy stored in electron donors (i.e., microbial feedstock) into biomass is described by η_bio_:

$$\eta_{\text{bio}}=Y_{\text{B}}\frac{\mathrm{\Delta H}{{}^{\circ}}_{B}}{\mathrm{\Delta H}{{}^{\circ}}_{\text{ED}}},$$ [3]

where Y_B_ is the biomass yield per mol electron donor consumed, ΔH°_B_ is the heat of combustion of bacterial biomass (i.e., stored energy in biomass) taken as 20 MJ · kg-dw^−1^ based on experimental result (71), and ΔH°_ED_ is as previously defined in Eq. **2**. Y_B_ values were taken from a recent study (25). For lower and upper bounds of η_bio_, we took the 30th and 70th percentile of the values of each combination of electron donor and assimilation pathway.

### Biomass-to-Food Product Efficiency.

The final step of food-grade SCP production is the conversion of wet biomass into protein by discarding all other cellular components, which we associated with the energy efficiency η_filter_. We assume that the filtering process maintains 100% of the protein. We calculated η_filter_ as follows:

$$\eta_{\text{filter}}=\text{ρ}\frac{\mathrm{\Delta H}{{}^{\circ}}_{\text{P}}}{\mathrm{\Delta H}{{}^{\circ}}_{\text{B}}},$$ [4]

where ρ is the fraction of usable protein in biomass on a gram per gram cell dry weight basis, ΔH°_P_ is the heat of combustion of protein in kJ per gram protein, and ΔH°_B_ is as previously defined in Eq. **3**. ρ is taken from literature and falls within a range of 55 to 75% (11, 72). ΔH°_P_ is taken as 16.7 MJ · kg^−1^ (73). The energetic costs required to extract protein from biomass are included in the microbial cultivation energy, as described below. Note that η_filter_ does not appear in the feed production scenario, since, in that case, all cellular components are retained in the final product.

### Effective Electricity Use Efficiency, η*.

This represents the fraction of electricity that is used in the electrochemical process for the generation of the electron donor compound (i.e., microbial feedstock). The rest of the electricity produced is distributed among several supporting processes: DAC of CO_2_, provision of macronutrients for microbial cultivation, bioreactor operation, and biomass downstream processing.

We collected available information regarding the energetic demand of DAC of CO_2_ using multiple technologies. Dataset S1C shows all values, where 6 and 9 MJ · kg-CO_2_^−1^ are the 30th to 70th percentiles. We converted these values to represent energy demand per kg biomass. We assumed that all CO_2_ released from the bioreactor (e.g., from formate and methanol oxidation to provide cellular energy) is directly recycled without an additional energetic cost. In this case, the energy demand for CO_2_ capture is directly proportional to the carbon assimilated into the microbial biomass. As the weight fraction of carbon in CO_2_ is 27% and in biomass it is 48% [assuming biomass formula of CH_1.77_O_0.49_N_0.24_ (74)], we obtained an energy demand for CO_2_ capture between 11 and 16 MJ · kg-dw^−1^. As the combustion energy of biomass is 20 MJ · kg-dw^−1^, the normalized energy demand for CO_2_ capture, θ_DAC_, ranges between 0.5 and 0.8. Low-temperature solid-sorbent DAC methods may also capture water in humid climates at a stoichiometry of 2 to 5 mol H_2_O per mol CO_2_ (19). This water could contribute to the water requirements of the electrochemical and cultivation processes.

The energy requirement for the supply of macronutrients—ammonium, phosphate, and sulfate—was calculated, per kg of dry weight biomass, based on life cycle assessment literature and cell stoichiometry (Dataset S1D). The approximate stoichiometry for producing 1 kg of dw biomass was taken as 1.76 kg CO_2_ (calculated in Dataset S1C), 0.112 kg NH_3_ (12), 0.02 to 0.03 kg H_3_PO_4_ (75), and 0.01 kg H_2_SO_4_ (76). The largest energy input is for provision of green NH_3_ (29.6 to 39.7 MJ per kg NH_3_) (77). The total energy cost for the provision of nutrients accounts for 4.8 to 6.6 MJ · kg-dw biomass^−1^ (Dataset S1D). Normalizing these values by the energy of combustion of 1 kg biomass gives θ_nut_, which lies in the range of 0.24 to 0.33.

Energy demand for the operation of the bioreactor, mainly stirring and cooling, was calculated to lie in the range 7.7 to 15.6 MJ · kg-dw^−1^ (Dataset S1D and *Methods*). Such a wide range of values derived from the fermentation industry allowed to consider a broad variety of possible reactor configurations and the related stirring and cooling requirements. The latter might differ greatly based on the substrates used and their solubility, and while this was out of the scope of this study, the considered range encompassed possible variations in this sense. Normalizing the 7.7 to 15.6 MJ · kg-dw^−1^ by the energy of combustion of 1 kg biomass gives θ_bioreactor_, which lies in the range of 0.39 to 0.78.

The energy demand for biomass downstream processing (i.e., converting the wet biomass into the final product) was calculated differently for feed and food production. In the case of feed production, which includes centrifugation and spray drying, the energy demand was calculated to be 8.4 to 9.1 MJ · kg-dw^−1^ (Dataset S1D). The energy demand for food production, which includes the additional steps of bead milling and microfiltration, was calculated to be 10.5 to 21 MJ · kg-dw^−1^ (Dataset S1D). Normalizing these values by the energy of combustion of 1 kg biomass gives θ_dsp_, which lies in the range of 0.42 to 0.46 for feed production and 0.52 to 1.1 for food production.

We note that the overall energy demand for provision of macronutrients, bioreactor operation, and biomass downstream processing is 21 to 31 MJ · kg-dw^−1^ for feed production and 23 to 43 MJ · kg-dw^−1^ for food production, which generally agrees with a previous study reporting energy demand of 28 to 32 MJ · kg-dw^−1^ (78).

The effective electricity use efficiency η* is defined as the fraction of electrical energy used for electrochemistry out of the total amount used in all processes. In order to produce 1 kg biomass, the energy required for electrochemistry is ΔH°_B_/(η_ec_ x η_bio_) and for all other processes is ΔH°_B_ x (θ_DAC_ + θ_nut_ + θ_bioreactor_ + θ_dsp_). Therefore:

$$\eta^{*}=\frac{\mathrm{\Delta H}{{}^{\circ}}_{\text{B}}\times \eta_{\text{ec}}^{-1}{\times \eta }_{\text{bio}}^{-1}}{\mathrm{\Delta H}{{}^{\circ}}_{\text{B}}{\times \eta }_{\text{ec}}^{-1}{\times \eta }_{\text{bio}}^{-1}+\mathrm{\Delta H}{{}^{\circ}}_{\text{B}}\times \left(\theta_{\text{DAC}}+\theta_{\text{nut}}+\theta_{\text{bioreactor}}+\theta_{\text{dsp}}\right)}=$$

$$1/\left(1+\eta_{\text{ec}}\times \eta_{\text{bio}}\left(\theta_{\text{DAC}}+\theta_{\text{nut}}+\theta_{\text{bioreactor}}+\theta_{\text{dsp}}\right)\right),$$ [5]

### Overall Solar-to-Feed/Food Energy Efficiency.

The overall energy efficiency of the solar-to-feed/food process, η_scp,_ is calculated by the product of the above-defined efficiencies. For the production of feed, the formula is:

$$\eta_{\text{scp}}=\eta_{\text{pv}}\times \eta_{\text{ec}}\times \eta_{\text{bio}}\times \eta^{*}\text{.}$$ [6]

For the production of food, the extra step of microfiltration discards calorie-containing biomass. Hence, this additional factor is multiplied into the production chain, reducing the overall efficiency:

$$\eta_{\text{scp}}=\eta_{\text{pv}}\times \eta_{\text{ec}}\times \eta_{\text{bio}}\times \eta_{\text{filter}}\times \eta^{*}\text{.}$$ [7]

### Estimation of Error.

To estimate the confidence interval for the η_scp_ in each scenario, we used standard propagation of uncertainty assuming all variables are independent. The uncertainty of each independent variable was taken as the difference between the 30th and 70th percentiles in our collected datasets (as shown in Table 1). The final uncertainty is the square root of the sum of squares of each variable’s uncertainty times the partial derivative of η_scp_ with respect to it.

### Solar-to-Electricity Efficiency Correction Function.

We identified a statistically significant negative correlation between annual irradiance and solar farm energy efficiencies (*P* value < 0.0001, *n* = 628), probably reflecting the known fact that solar cells become less efficient at higher temperatures (32). We fitted a regression equation of irradiance to solar farm efficiency PV_R_, while systematically discarding outliers. This was performed iteratively, wherein each iteration data point for which the measured efficiency was different from the predicted one by more than 0.03 were discarded, and the regression was recalculated. We found the regression function to be:

$${\mathrm{PV}}_{\text{R}}=\;0\text{.}077\text{– I}/\left(59000{\text{kWh·m}}^{-2}{\text{·y}}^{-1}\right),$$ [8]

where I is the annual irradiance in the range of 700 to 2,700 kWh · m^−2·^ y^−1^. We divided PV_R_ by median solar farm efficiency to quantify the deviation from median as a function of irradiance. The resulting equation is the solar correction function, f_C_:

$$f_{\text{C}}=\;1\text{.}6\text{– I}/\left(2800{\text{kWh·m}}^{-2}{\text{·y}}^{-1}\right).$$ [9]

At low irradiance (I < 1,680 kWh · m^−2^ · y^−1^), f_C_ is greater than 1, and at high irradiance, f_C_ is less than 1.

### Yield of Food Energy and Protein.

The yield of nutritional calories per land area and time, Y_cal_, is given by:

$$Y_{\text{cal}}=\text{I}\times f_{\text{C}}\times \eta_{\text{scp}},$$ [10]

where I is irradiance ranging from 700 to 2,700 kWh m^−2^ y^−1^_,_ η_scp_ is as in Eq. **6**, and f_C_ is as in Eq. **7**.

In the case of food production, the energy contained in the final product, Y_cal_, reflects only the energy in the protein fraction of the biomass. Hence, the SCP system yield in terms of protein mass for food production, Y_prot_, is determined by dividing the food energy yield by the energy content per unit protein:

$$Y_{\text{prot}}=\frac{Y_{\text{cal}}}{\mathrm{\Delta H}{{}^{\circ}}_{\text{P}}},$$ [11]

where Y_prot_ is the yield of protein in g · m^−2^ · y^−1^ and $\mathrm{\Delta H}{{}^{\circ}}_{\text{P}}$ is as defined in Eq. **4**.

#### Yield of food/feed energy for SCP grown on sucrose extracted from sugar beet.

The production of SCP from sucrose extracted from sugar beet requires two plots of land. In one plot, sugar beet is cultivated. In the other, PV arrays are placed to generate the electricity needed for the extraction of sucrose and cultivation of microbes.

Upon derivation (described in the *SI Appendix*), we obtain an energy yield of SCP produced via sugar beet as follows:

$$Y_{SB-SCP}=\frac{Y_{scal}\times \eta_{\text{bio}}\times \eta_{\text{filter}}}{\frac{\;Y_{scal}\left(\left(\theta_{\text{nut}}+\theta_{\text{bioreactor}}+\theta_{\text{dsp}}\right)\times \eta_{\text{bio}}+\theta_{\text{sx}}+\theta_{\text{scult}}\right)}{I\times \eta_{\text{pv}}\times f_{c}\;}+1}.$$ [12]

Y_scal_ is the energetic yield of sucrose derived from sugar beet, which was calculated as 6.5 kg · m^−2^ · y^−1^ (Dataset S1E) multiplied by 16%, the characteristic extractable sucrose content per fresh weight sugar beet (79), and converted to units of energy (considering that 16.7 MJ · kg-sucrose^−1^) to give 4.8 kWh · m^−2^ · y^−1^. θ_scult_, the energy cost of sugar beet cultivation (11 to 28 GJ · ha^−1^) normalized by sucrose yield and the energy of combustion of sucrose (16.7 MJ · kg^−1^), ranges between 0.13 and 0.19 (Dataset S1H). θ_sx_ is the energy cost of sucrose extraction (0.2 MJ per kg-sugar beet (80) which converts to 1.25 MJ · kg-sucrose^−1^) normalized to the combustion energy of sucrose, which results in 0.07. θ_nut_, θ_bioreactor_, θ_dsp_, and f_C_ are as defined above. We note that the contribution of and thus the energy required to extract sucrose is negligible compared with the energy required for microbial cultivation. Note that in the case of feed production, the term $\eta_{\text{filter}}$ is omitted from the numerator, which increases the overall yield.

To calculate the protein yield of SCP produced via sugar beet in units of mass per land area and time, we divide Y_SB-SCP_ by the combustion energy of protein (16.7 MJ · kg^−1^).

#### Protein consumption.

The average kcal intake is 2,150 kcal per person per day (48), 15% of which should be protein (81). Hence, ≈80 g protein is the assumed protein mass consumed per person per day, or about 30 kg · y^−1^.

## Supplementary Material

Supplementary File

Supplementary File

Supplementary File

## Data Availability

All study data are included in the article and/or supporting information.

## Change History

January 10, 2022: Dataset S1 has been updated.
