# Supplementary material for: Photovoltaic-driven microbial protein production can use land and sunlight more efficiently than conventional crops
Source: Proc Natl Acad Sci U S A. 2021 Jun 21;118(26):e2015025118. doi: 10.1073/pnas.2015025118 (PMC8255800; doi:10.1073/pnas.2015025118)
Supplement: Supplementary File [file pnas.2015025118.sapp.pdf]

## Supplementary Information for

# Photovoltaic-driven microbial protein production can use land and sunlight more efficiently than conventional crops

Dorian Leger[1], Silvio Matassa[2], Elad Noor[3], Alon Shepon[4,5], Ron Milo[3], Arren Bar-Even[1]

[1]Max Planck Institute of Molecular Plant Physiology, Germany

[2]Department of Civil, Architectural and Environmental Engineering, University of Naples Federico II, Italy

[3]Department of Plant and Environmental Sciences, Weizmann Institute of Science, Israel

[4]Department of Environmental Studies, The Porter School of the Environment and Earth Sciences, Tel Aviv University, Israel

[5]The Steinhardt Museum of Natural History, Tel Aviv University, Israel

* Corresponding author: dorian.dleger@gmail.com

**This PDF file includes:**

Supplementary text

Figure S1

Figure S2

Figure S3

Tables S1

SI References

**Other Supplementary Materials:**

Dataset S1

Dataset S2

## Role of carbon in the electrochemical and biological processes

To provide a broad perspective on the properties of different electrochemical and biological processes, we considered the production of three electron donors (hydrogen, methanol, and formate) to serve as the energy sources for biomass growth. For the production of all of the three electron donors, water is first split and oxidized at an anode to provide electrons and oxygen ($O_2$) to the subsequent processes. The entry point of $CO_2$ (obtained by direct air capture of $CO_2$) in the production chain depends on the choice of electron donor, as summarized in Figure S1 and Figure S2. If hydrogen serves as the electron donor (A), it is produced at the cathode (during water electrolysis) and supplied alongside $CO_2$ and $O_2$ to the bioreactor to feed autotrophic aerobic hydrogenotrophs. If formate is the electron donor (B), $CO_2$ is reduced at a specialized cathode during water electrolysis, and the resulting formate ($HCOO^-$) is supplied alongside $O_2$ to the bioreactor to feed aerobic formatotrophs. If methanol is the electron donor (C), the hydrogen derived from water electrolysis is instead used to reduce the $CO_2$ to methanol ($H_3COH$) in a separate catalytic reactor, which is then supplied alongside $O_2$ to the bioreactor to cultivate aerobic methanotrophs.

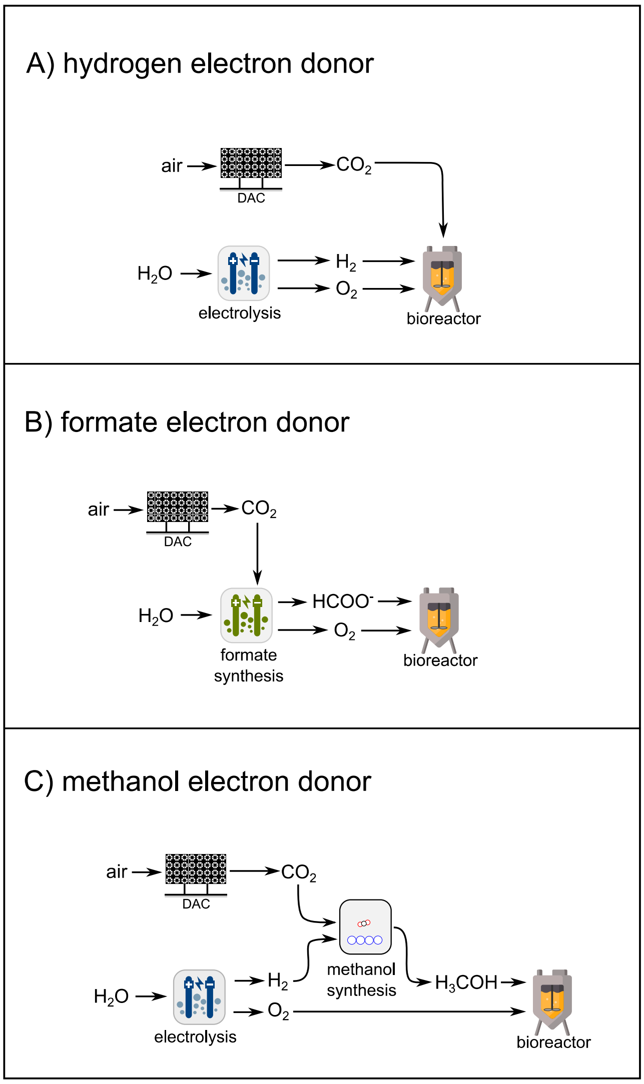


**Figure S1. Schematic representation of three processes for electron donor production and utilization.** In each case water serves as the initial source of electrons, and its oxidation yields dioxygen gas. Carbon dioxide, which is obtained by direct air capture of $CO_2$ (DAC), enters the production chain differently according to the choice of electron donor. A) For production of hydrogen to feed hydrogenotrophs, the $CO_2$ is supplied to the bioreactor. B) For production of formate to feed formatotrophs, the $CO_2$ is supplied to the cathode of an electrochemical cell. C) For production of methanol to feed methanotrophs, a two-step reaction occurs, where first water is split to yield hydrogen, and then hydrogen is combined with a supply of $CO_2$ in a separate catalytic reactor to produce methanol.

I) hydrogen serving as the electron donor

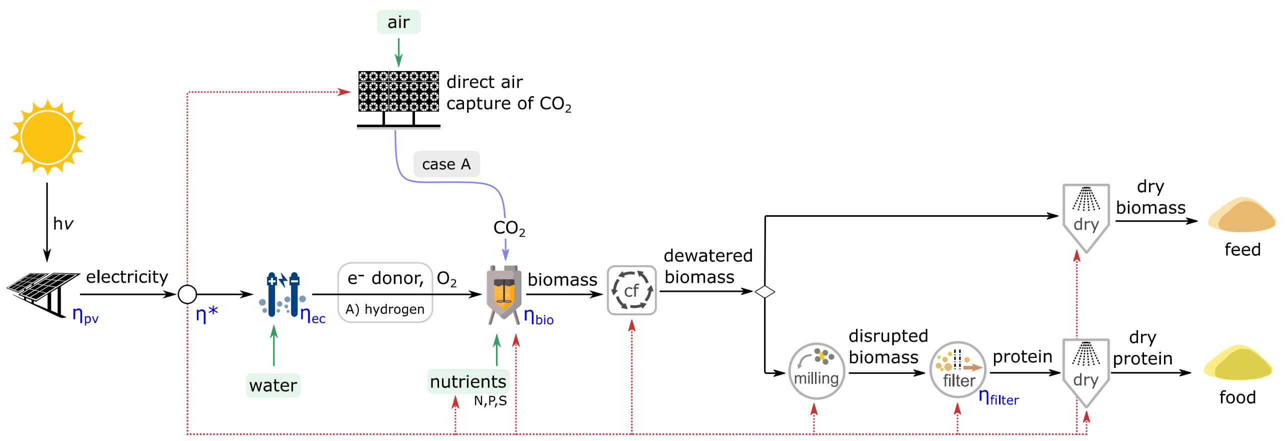


II) formate or methanol serving as the electron donors

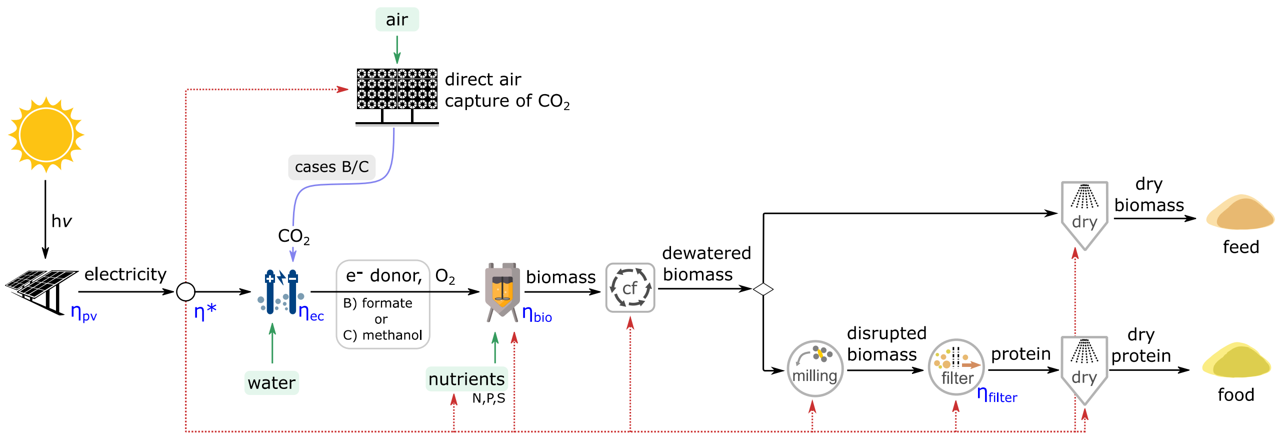


**Figure S2. Schematic representation of energy transfer during production of single cell protein from solar energy.** This figure is a more detailed view of the processes depicted in the main-text Figure 1. Each conversion step is associated with an energetic efficiency, η. The effective electricity use efficiency, η*, corresponds to the fraction of electricity used for electrosynthesis of the electron donor. The rest of the electricity (dashed red arrows) is distributed among supporting processes, including direct air capture of $CO_2$, provision of macronutrients, bioreactor operation, and biomass downstream processing. The upper panel (I) shows case A, where hydrogen serves as the electron donor. In case A, $CO_2$ enters the production chain at the bioreactor. The lower panel (II) shows cases B/C, where formate or methanol serve as the electron donors. In cases B/C, $CO_2$ enters the production chain at the electrochemical units. For both panels, two production scenarios are depicted based on the desired final product. In the production of feed for animals, a relatively simple downstream processing is required, which involves only the removal of water by centrifugation (cf), and spray-drying (dry). For the production of food for humans, two additional steps, bead-milling and microfiltration, are required to reject nucleic acids from the final product. Since biomass is discarded in the filtration step, food production has an additional energy efficiency factor denoted by $\eta_{filter}$.

**Energy efficiency of crops**

Here, the energy efficiency of crops is defined as the combustible energy in the edible portion of the crop divided by the input annual solar energy. To calculate this efficiency, granular datasets are required which enable the association of a yield value to an input irradiance. The calculations are presented in Dataset S2. The energy efficiencies of soybean, maize, and sugar beet were assessed using three crop datasets: The United States Department of Agriculture (USDA), a largescale Corteva cultivar trial in Bulgaria, and the Indian Ministry of Agriculture and Farmer's Welfare. Solar data was sourced from the Global Horizontal Irradiance (GHI) dataset of National Renewable Energy Laboratory [1]. The average irradiance for each region in the crop datasets was obtained using the GIS tool QGIS (version 3.10.2), by associating the annual irradiance information as a raster layer to a vector layer with polygons representing the regions. For each crop, the energy efficiency of converting solar energy to edible biomass was calculated by converting the mass yields of crops (for a given region and given year) to an equivalent energetic content, and then dividing by the irradiance incident on the given land area in the given year. The FAO conversion factors for edible mass of crop to calorie and protein are found in Dataset S1E. The energy efficiency of maize silage was calculated in the same way, while also applying a dry matter content of 23% to the yields (because the harvested yield were not reported on dry matter basis for silage) [2], and then converting to energetic content using the factor 4.2 kcal g-dm$^{-1}$ [2]. The solar to protein energy efficiency of all crops was calculated by applying a protein content factor to the mass yield (Dataset S1E), and then multiplying the protein content by 16.7 kJ g$^{-1}$, and subsequently dividing by the incident solar energy.

**Soybean allocation of biomass**

The soybean harvest index is ≈0.40 [3, 4], where harvest index = grain dw / shoot dw. Note that the shoot is defined as the combination of grain and stover. The total plant biomass is the sum of root and shoot. The soybean root to shoot mass ratio is 0.17 [5], otherwise expressed as root = 0.17 x shoot. This latter equation and the harvest index above can be used to solve for the fraction of biomass allocated to edible grain over the total plant biomass (i.e., the fraction of grain/ [shoot + root]), resulting in the value of edible grain / total biomass = 0.34.

**Water requirements for electron donor production**

Water supplies the electrons required to produce the electron donors considered in this study. Three production processes were considered depending on the chosen electron donor (i.e., A) hydrogen, B) formate, C) methanol). The water required for electrochemistry per unit biomass is estimated for each case using the median energy efficiencies by which electron donors are converted into biomass energy, considering the most efficient metabolic pathways in each case (denoted here as $\eta_{\mathrm{bio}}^{\mathrm{e-\ donor}}$).

A) When hydrogen is the electron donor, 1 mol of water is needed per mol hydrogen:

$$2\ H_2O \rightarrow 2\ H_2 + O_2$$

Using $\eta_{\mathrm{bio}}^{\mathrm{H}} \approx 0.345$ we can calculate that production of 1 kg biomass, which is equivalent to 20 MJ combustible energy, requires 57 MJ of hydrogen (20 MJ/0.345 ≈ 57 MJ). As the lower heating value combustion energy (LHV) of hydrogen is 0.26 MJ/mol, then 1 kg dw biomass requires ≈220 mols $H_2$. Hence 220 mols of $H_2O$ are required, which, using water's molar mass of 18 g/mol, is equivalent to ≈4 kg-$H_2O$ kg-dw-biomass$^{-1}$.

B) When formate is the electron donor, the water requirement is 1 mol per mol formate:

$$2\ H_2O + 2\ CO_2 \rightarrow 2\ HCOOH + O_2$$

Using $\eta_{\mathrm{bio}}^{\mathrm{F}} \approx 0.46$ (assuming the serine cycle is used), we get that 1 kg biomass requires ≈43.5 MJ of formate (20 MJ/0.46 ≈ 43.5 MJ). As the LHV for formate is 0.245 MJ/mol, then 1 kg dw biomass requires ≈177 mols formate. Hence 177 mols $H_2O$ are required, which is equivalent to ≈3.2 kg-$H_2O$ kg-dw-biomass$^{-1}$.

C) When methanol is the electron donor, 2 mols of water are needed per mol methanol (as some water is reformed in the second reaction):

$$3\ H_2O \rightarrow 3\ H_2 + 1.5\ O_2 \quad \text{followed by} \quad 3\ H_2 + CO_2 \rightarrow H_3COH + H_2O$$

Using $\eta_{\mathrm{bio}}^{\mathrm{M}} \approx 0.44$ (assuming RuMP cycle is used), we get that 1 kg biomass requires ≈45.5 MJ of methanol (20 MJ/0.44 ≈ 45.5 MJ). As the LHV for methanol is 0.71 MJ/mol, then 1 kg dw

biomass requires ≈64 mols methanol. Hence 128 mols $H_2O$ are required, which is equivalent to ≈2.3 kg-$H_2O$ kg-dw-biomass$^{-1}$

**SCP production cost.** We performed a preliminary estimate of the production costs associated with SCP by relying upon recently available literature studies [6, 7]. These estimates were updated and adapted by considering the capital and operational costs linked to renewable energy-powered hydrogen electrolysis, methanol and formate production, as well as $CO_2$ capture through DAC and green ammonia synthesis. The estimates resulted in a range of potential production costs for hydrogen-, methanol- and formate-based SCP for feed and food purposes (Dataset S1G). Only the metabolic pathways showing the higher overall energy efficiency of the solar to feed/food process ($\eta_{scp}$) for each electron donor were considered for the potential production of SCP for feed or food.

Generalized capital and operational costs for SCP production were considered valid for all different SCP products. To avoid the potential bias linked to the specific SCP fermentation equipment needed for production from hydrogen, methanol and formate, capital investments for gas-based SCP production plants were used as the reference value (see “value high” in Dataset S1G) for all different SCP systems [7]. The main capital investments for equipment and infrastructures included the fermentation and post-processing steps of SCP plants producing between 25,000 and 108,000 ton-dw-biomass y$^{-1}$. The share of production costs related to the latter capital investments was calculated using the annuity method, by considering a capital cost of $50 to $251 million, an equipment lifetime and repayment period of 25 years, interest rates of 3% to 8% and loan repayments on an annual basis [6]. In order to account for the higher capital investments needed for the production of SCP as human food, which would require also the bead milling and filtration steps to purify proteins, the capital investment was conservatively increased by 20% and 40% for the low and high values, respectively. The annuity of capital costs for the fermentation plant were calculated to be $0.11 to $0.22 kg-dw-biomass$^{-1}$ for SCP as animal feed and $0.14 to $0.30 kg-dw-biomass$^{-1}$ for SCP as human food. The general operational costs linked to the supply of nitrogen through green ammonia synthesis, including both capital and operational expenses, were extrapolated from Osman et al. [8], and contributed to SCP production costs between $0.06 and $0.10 kg-dw-biomass$^{-1}$. Other production costs included the provision of phosphorus through phosphoric acid production ($0.03 to $0.06 kg-dw-biomass$^{-1}$) [6] and the energy consumption of SCP fermentation and post-processing. The latter costs were estimated by considering a range of $0.05 to $0.10 kWh$^{-1}$ for renewable energy [6], and applying these to the energy demand of SCP production estimated in Dataset S1D for animal feed and human food. Operational costs for SCP fermentation ranged between $0.11 and $0.43 kg-dw-biomass$^{-1}$, while costs for post-processing were $0.12 to $0.25 kg-dw-biomass$^{-1}$ for animal feed and $0.15 to $0.58 kg-dw-

biomass$^{-1}$ for human food. Potential overheads, including costs for the supply of other macro- and micro-nutrients, consumables and labour, were assumed as equal to the annuity of capital expenses for the fermentation plant for animal feed ($0.11 to $0.22 kg-dw-biomass$^{-1}$) or human food ($0.14 to $0.30 kg-dw-biomass$^{-1}$) production [6].

Both capital and operational costs linked to the supply of $CO_2$ through renewable energy-powered DAC were extrapolated from Keith et al. [9], while the costs for the provision of renewable electron donors were specifically estimated for each SCP production process (i.e., from hydrogen, methanol or formate). For hydrogen-based SCP (Calvin cycle), projected 2030 levelized costs of industrial-scale solar-powered electrolytic $H_2$ production associated with pressure vessel $H_2$ storage systems were considered [10]. The final total estimated hydrogen-based SCP production costs were $1.9 to $3.4 kg-dw-biomass$^{-1}$ ($2.5 to $6.2 kg-protein$^{-1}$) for animal feed and $1.9 to $3.8 kg-dw-biomass$^{-1}$ ($2.6 to $7.0 kg-protein$^{-1}$) for human food. Methanol-based SCP (RuMP cycle) production costs were calculated by considering the levelized cost of industrial-scale methanol production from renewable energy and $CO_2$ from DAC [11]. The total estimated methanol-based SCP production costs were $1.7 to $3.9 kg-dw-biomass$^{-1}$ ($2.3 to $7.2 kg-protein$^{-1}$) for animal feed and $1.8 to $4.4 kg-dw-biomass$^{-1}$ ($2.3 to $7.9 kg-protein$^{-1}$) for human food. Finally, formate-based SCP (serine cycle) production costs considered the cost of renewable formate production through electrochemical reduction of $CO_2$ captured through DAC [12]. The total estimated formate-based SCP production costs were $3.5 to $15.1 kg-dw-biomass$^{-1}$ ($4.7 to $27.4 kg-protein$^{-1}$) for animal feed and $3.5 to $15.5 kg-dw-biomass$^{-1}$ ($4.7 to $28.2 kg-protein$^{-1}$) for human food. Dataset S1G summarizes the final range of production costs calculated for each SCP technology, and compares them with current market prices of conventional animal feed and alternative human food products.

**Appendix Table S1.** Production costs of the best performing SCP technologies investigated in this study in terms of dry weight SCP biomass and protein. As a means of comparison, the current market price of soybean meal and fishmeal are used as reference for conventional animal feed products, while several alternative human food protein products are displayed.

| Electron donor | Metabolic pathway | Protein content | Production cost for animal feed | | Production cost for human food | |
|---|---|---|---|---|---|---|
| | | | $ kg-dw-biomass$^{-1}$ | $ kg-protein$^{-1}$ | $ kg-dw-biomass$^{-1}$ | $ kg-protein$^{-1}$ |
| Hydrogen | Calvin cycle | 55-75% | 1.9-3.4 | 2.5-6.2 | 1.9-3.8 | 2.6-7.0 |
| Methanol | RuMP cycle | | 1.7-3.9 | 2.3-7.2 | 1.8-4.4 | 2.3-7.9 |
| Formate | Serine cycle | | 3.5-15.1 | 4.7-27.4 | 3.5-15.5 | 4.7-28.2 |
| **Animal feed products** [a] | | | **Market price** | | | |
| | | | $ kg-dw-biomass$^{-1}$ | | $ kg-protein$^{-1}$ | |
| Soybean meal | | 30-40% | 0.4 | | 0.9-1.2 | |
| Fishmeal | | 55-65% | 1.5 | | 2.3-2.7 | |
| **Human food products** [b] | | | **$ kg-protein$^{-1}$** | | | |
| Soy protein | | ~75% | 2 | | | |
| Pea protein | | ~75% | 5 | | | |
| Whey protein | | ~90% [c] | 7 | | | |
| Mycoprotein | | ~47% | 13 | | | |
| Insect protein | | <25% | 41 | | | |
| Cultured meat | | - | 300 | | | |

[a] Data from Matassa et al., 2020 [13]

[b] Data from Bashi et al., 2019 [14]

c Data from Foegeding et al., 2011 [15]

## Yield of food/feed energy for SCP grown on sucrose extracted from sugar beet

When sugar beet serves as the source of carbon and energetic feedstock for microbial growth, land must be partitioned towards two uses (Figure S3): sugar beet land ($Land_{SB}$) and photovoltaic solar farm land ($Land_{PV}$). $Land_{SB}$ provides the feedstock, while $Land_{PV}$ generates electricity for supporting processes, such as sucrose extraction, bioreactor operation, and biomass downstream processing.

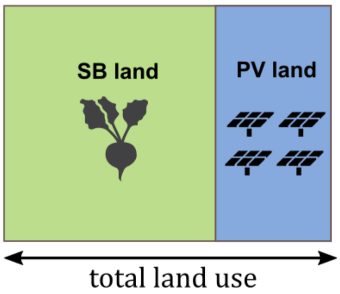


**Figure S3. Depiction of land use in SCP produced from sugar beet.** For SCP from sugar beet, land is allocated to two different uses, cultivation of sugar beet (SB land) and generation of electricity from photovoltaics (PV land). The yield of the land must account for sum of all land used (total land use).

When considering an SCP food product, we defined the caloric yield as the combustion energy of protein that can be obtained per unit of land.

$$Y_{SB-SCP} = \frac{\text{Energy yield in protein}}{\text{Total land}} = \frac{\text{Energy yield in protein}}{\text{Land}_{SB} + \text{Land}_{PV}} \quad \textbf{[1]}$$

The numerator in equation (1) is calculated by considering the sucrose energy yielded per unit of sugar beet land ($Y_{scal}$), multiplied by the energetic efficiency of biomass growth on sucrose ($\eta_{bio}$), multiplied by the energy efficiency of extracting protein from the whole biomass $\eta_{filter}$.

$$\text{Energy yield in protein} = Y_{scal} \times \eta_{bio} \times \eta_{filter} \quad \textbf{[2]}$$

Next, the $\text{Land}_{PV}$ requirement can be expressed as the total supporting electrical energy required divided by the electrical energy produced per unit land. Hence, $\text{Land}_{PV}$ is:

$$\text{Land}_{PV} = \frac{\text{total sup. energy required}}{\text{electricity generated per unit land}} \quad \textbf{[3]}$$

Total supporting energy can be broken into two parts. There is energy required to process biomass, and energy to cultivate sugar beet and process sucrose.

First, the energy required to process biomass is equal to the amount of biomass energy yielded (given by $Y_{scal} \times \eta_{bio}$) multiplied by the supporting energy requirements per unit biomass energy for: nutrient provision ($\theta_{nut}$), bioreactor operation ($\theta_{bioreactor}$), and downstream processing ( $\theta_{dsp}$). See Methods for definitions of these factors.

$$\text{Supporting energy for biomass} = Y_{scal} \times \eta_{bio} \times (\theta_{nut} + \theta_{bioreactor} + \theta_{dsp}) \quad \textbf{[4]}$$

Supporting energy requirements for cultivating sugar beet ($\theta_{scult}$) and extracting sucrose ($\theta_{sx}$) per unit sucrose energy is given by:

$$\text{Sup. energy for SB cultivation and sucrose exraction} = Y_{scal} \times (\theta_{sx} + \theta_{scult}) \quad \textbf{[5]}$$

Hence, the sum of the (4) and (5) gives the total supporting energy required.

$$\text{Total sup. energy required} = Y_{scal} \times (\eta_{bio} \times (\theta_{nut} + \theta_{bioreactor} + \theta_{dsp}) + \theta_{sx} + \theta_{scult}) \quad \textbf{[6]}$$

Next, $\text{Land}_{PV}$ generates the following amount of electricity:

$$\text{Electricity generated per unit PV land} = I \times \eta_{pv} \times f_c \quad \textbf{[7]}$$

Where I is the local irradiance, $\eta_{pv}$ is the conversion efficiency of solar to electrical energy, and $f_c$ is the solar correction function. Each of these terms is defined in Methods.

Hence, the $\text{Land}_{PV}$ required (Eq. **[3]**) can be solved by dividing Eq. **[6]** by Eq. **[7]**.

$$\text{Land}_{PV} = \frac{Y_{scal} \times (\eta_{bio} \times (\theta_{nut} + \theta_{bioreactor} + \theta_{dsp}) + \theta_{sx} + \theta_{scult})}{I \times \eta_{pv} \times f_c} \quad \textbf{[8]}$$

Next, we can use Eq. **[2]** and Eq. **[8]** to substitute into Eq. **[1]**.

$$Y_{SB-SCP} = \frac{\text{Energy yield in protein}}{\text{Land}_{SB} + \text{Land}_{PV}} = \frac{Y_{scal} \times \eta_{bio} \times \eta_{filter}}{\text{Land}_{SB} + \frac{Y_{scal} \times (\eta_{bio} \times (\theta_{nut} + \theta_{bioreactor} + \theta_{dsp}) + \theta_{sx} + \theta_{scult})}{I \times \eta_{pv} \times f_c}}$$

To solve this equation we set $\text{Land}_{SB}$ equal to 1 $m^2$, in which case the denominator is 1 unit of $\text{Land}_{SB}$ plus the proportional requirement of $\text{Land}_{PV}$. Hence, the food energy yield of SB-SCP is given by:

$$Y_{SB-SCP} = \frac{Y_{scal} \times \eta_{bio} \times \eta_{filter}}{1 + \frac{Y_{scal} \times (\eta_{bio} \times (\theta_{nut} + \theta_{bioreactor} + \theta_{dsp}) + \theta_{sx} + \theta_{scult})}{I \times \eta_{pv} \times f_c}} \quad \textbf{[9]}$$

Note that in the case of feed production the term $\eta_{filter}$ is omitted from the numerator, which increases the overall yield.

## References


1. NREL. National Renewable Energy Laboratory. https://maps.nrel.gov/nsrdb-viewer/ (2021).
2. Wei, M., Chen, Z., Wei, S., Geng, G. & Yan, P. Comparison among methods of effective energy evaluation of corn silage for beef cattle. *Asian-Australas J Anim Sci* **31**, 851-858 (2018).
3. Jiang, W., Liu, X., Xiukang, W. & Yin, Y. Characteristics of Yield and Harvest Index, and Evaluation of Balanced Nutrient Uptake of Soybean in Northeast China. *Agronomy Journal* **9**, 310 (2019).
4. Krisnawati, A. & Adie, M. Variability of Biomass and Harvest Index from Several Soybean Genotypes as Renewable Energy Source. *Energy Procedia* **65** (2015).
5. Ordóñez, R.A. et al. Root to shoot and carbon to nitrogen ratios of maize and soybean crops in the US Midwest. *European Journal of Agronomy* **120**, 126130 (2020).
6. Pikaar, I. et al. Decoupling Livestock from Land Use through Industrial Feed Production Pathways. *Environmental Science & Technology* **52**, 7351-7359 (2018).
7. García Martínez, J.B. et al. Potential of microbial protein from hydrogen for preventing mass starvation in catastrophic scenarios. *Sustainable Production and Consumption* **25**, 234-247 (2021).
8. Osman, O., Sgouridis, S. & Sleptchenko, A. Scaling the production of renewable ammonia: A techno-economic optimization applied in regions with high insolation. *Journal of Cleaner Production* **271** (2020).
9. Keith, D.W., Holmes, G., St. Angelo, D. & Heidel, K. A Process for Capturing CO2 from the Atmosphere. *Joule* **2**, 1573-1594 (2018).
10. Mallapragada, D.S., Gençer, E., Insinger, P., Keith, D.W. & O'Sullivan, F.M. Can Industrial-Scale Solar Hydrogen Supplied from Commodity Technologies Be Cost Competitive by 2030? *Cell Reports Physical Science* **1** (2020).
11. Bos, M.J., Kersten, S.R.A. & Brilman, D.W.F. Wind power to methanol: Renewable methanol production using electricity, electrolysis of water and CO2 air capture. *Applied Energy* **264** (2020).
12. Ramdin, M. et al. High-Pressure Electrochemical Reduction of CO2 to Formic Acid/Formate: Effect of pH on the Downstream Separation Process and Economics. *Industrial & Engineering Chemistry Research* **58**, 22718-22740 (2019).
13. Matassa, S. et al. Upcycling of biowaste carbon and nutrients in line with consumer confidence: the "full gas" route to single cell protein. *Green Chemistry* **22**, 4912-4929 (2020).
14. Bashi, Z., McCullough, R., Ong, L. & Ramirez, M. Alternative proteins: The race for market share is on. *McKinsey & Company* (2019).
15. Foegeding, E.A., Luck, P. & Vardhanabhuti, B. in Encyclopedia of Dairy Sciences (Second Edition). (ed. J.W. Fuquay) 873-878 (Academic Press, San Diego; 2011).
